# Effects of dispersed oil on reproduction in the cold water copepod *Calanus finmarchicus* (Gunnerus)

**DOI:** 10.1002/etc.2273

**Published:** 2013-07-16

**Authors:** Anders Johny Olsen, Trond Nordtug, Dag Altin, Morten Lervik, Bjørn Henrik Hansen

**Affiliations:** † Norwegian University of Science and Technology, Department of BiologyRealfagbygget, Trondheim, Norway; ‡ SINTEF Materials and Chemistry, Marine Environmental TechnologyTrondheim, Norway; § BioTrix, TrondheimNorway

**Keywords:** *Calanus finmarchicus*, Copepod, Dispersed oil, Ecotoxicology, Reproduction effects

## Abstract

Following a 120-h exposure period to 3 concentrations of oil dispersions (0.022 mg L^−1^, 1.8 mg L^−1^, and 16.5 mg L^−1^, plus controls) generated from a North Sea crude oil and a subsequent 21-d recovery, mortality, and several reproduction endpoints (egg production rates, egg hatching success, and fraction of females participating in reproduction) in *Calanus finmarchicus* were studied. Concentration-dependent mortality was found during exposure, averaging to 6%, 3%, 15%, and 42% for the controls and 3 exposure levels, respectively. At the start of the recovery period, mean egg production rates of surviving females from the highest concentrations were very low, but reproduction subsequently improved. In a 4-d single female reproduction test starting 13 d postexposure, no significant differences in egg production rates or hatching success were found between reproducing control and exposed copepods. However, a significantly lower portion of the surviving females from the highest exposure participated in egg production. The results indicate that although short-term exposure to oil-polluted water after an oil spill can induce severe mortality and temporarily suspend reproduction, copepods may recover and produce viable offspring soon after exposure. The results might imply that for *C. finmarchicus* populations, the impact from short-term exposure to an oil spill might be predicted from acute mortality and that delayed effects make only a limited contribution to population decrease.

## INTRODUCTION

Oil exploration and production in the North Sea and the Norwegian Sea have been occurring for decades, and exploration is moving rapidly toward Arctic areas, including the Barents Sea and Chukchi Sea. A proper understanding of the fate and effects of potential oil spills in these areas is required to provide reliable and relevant oil spill response actions and contingency planning. The results from field studies and laboratory tests' protocols and numerical modeling have been applied to reach this goal.

For organisms living in the pelagic environment, dissolved oil components are known to be a main cause of toxic effects of petrogenic oils, and several tests have aimed to evaluate the effect of the water-soluble fraction or the water-accommodated fraction of many oils on a large array of test organisms. Oil spilled into the sea, however, may form micro-sized oil droplets in the water column when exposed to turbulence created by surface wave action, and the process is enhanced by adding chemical dispersants. The droplets may act as a source for further dissolution of oil components into the water; in addition, they contain low–water-soluble components (alkanes, decalins, and heavier polycyclic aromatic hydrocarbons [PAHs]) that may become bio-available through ingestion by micro- or mesoplankton species. Ingestion of particulate oil has been observed in filter-feeding copepods 1,2 and tunicates 4.

Copepods generally dominate the mesoplankton communities of the world's oceans, both in terms of production and standing biomass 5–6. They are a crucial link in the food chain for energy and matter, positioned between the pelagic primary production (microalgae) and higher trophic-level organisms, including several commercial fish species. They have also received considerable attention as targets in oil pollution impact test bioassays. In particular, the effects of single PAHs on copepods have been reported extensively 7–14, and exposure studies with oil have been performed 1–17. The majority of oil exposure studies involving copepods have focused on short-term exposure and acute toxicity (narcosis). Only a few of these studies have investigated the impact of oil or PAH on copepod reproduction 16,18. No studies have assessed the long-term effects of acute oil exposure.

The *Calanus* genera represents the dominant zooplankton species in sub-Arctic and Arctic waters 5, and *Calanus finmarchicus* (Gunnerus) is a key element of the sub-Arctic food chain. The species grazes on phytoplankton during algal bloom in the spring and summer and functions as an important energy and lipid source for fish and fish larvae 6–20. The reproductive biology of *C. finmarchicus* has been studied comprehensively in the field 21 and in laboratory cultures 22.

Recently, our research group developed an experimental system for continuous generation of oil dispersions that are stable over time, with defined oil droplet size ranges and good concentration control 23. This standardization increases the applicability of the test results in environmental risk assessment, and the resulting data can be properly parameterized for the use in models. To date, the system has been used to assess effects of oil dispersions on copepod filtering capacity and survival 1, and we have shown that *C. finmarchicus* can readily filter oil droplets 1–2.

The purpose of the present study was to evaluate the potential impact of sublethal episodic exposure to dispersed crude oil on *C. finmarchicus* reproduction.

## MATERIALS AND METHODS

### Test equipment and procedures

#### Copepods

*Calanus finmarchicus* from the continuous laboratory culture at the Norwegian University of Science and Technology/SINTEF SeaLab was used for the experiments. The culture is routinely kept at approximately 10 °C in a 16:8-h night:day cycle and continuously fed a mixture of 3 marine microalgae (*Rhodomonas baltica* Karsten, *Dunaliella tertiolecta* Bucher, and *Isochrysis galbana* Parke) ad libitum. Further details on the culturing is given in Hansen et al. 24. Young but presumably fertilized females were collected for experiments from a subset of the running culture. To increase mating probability, newly moulted adults were allowed a few days in the culture before being collected and transferred to the exposure system.

#### Experimental setup

The experimental setup included the following main subsystems: an oil droplet generation system and a computerized distribution unit 23; a set of exposure tanks (n = 4), each with a custom-made computerized mixing device; a set of recovery tanks (n = 12); and a set of custom-made egg-laying chambers (n = 120). The whole setup was installed in a temperature-controlled room (10 ± 0.5 °C). A 16:8-h light:dark cycle with simulated dusk and dawn similar to that used for the cultures was applied throughout the experimental period of 26 d. The seawater (34.5‰) used for the experiment was collected at 70 meters depth in Trondheimsfjorden (Norway), sand filtered, temperature adjusted to 10 ± 0.2 °C and equilibrated with air. Before application in an experiment, a final inline filtration to 1 µm was included (Cuno Aqua-Pure water filter system). During the experiment, the copepods were fed the same mixture of microalgae as the running cultures, with each algae species amounting to one-third on a carbon basis. Mixed algae stock solution was exchanged with fresh stock on a daily basis and continuously added by a peristaltic pump to give a concentration in the exposure water of nominally 2 mg wet weight L^−1^ (∼300 µg C L^−1^) for all groups throughout the experiment. Algae were supplied from in-house running cultures, and concentrations with few exceptions were verified daily by particle counting.

#### Time schedule

**Figure 1:**
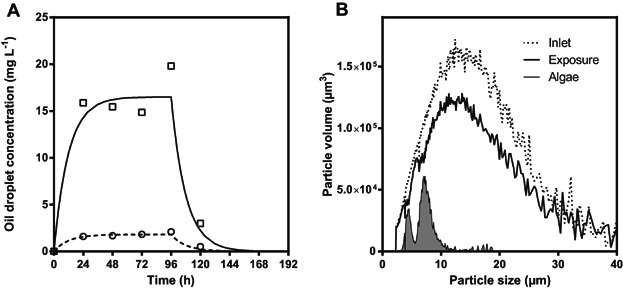
(A) Theoretical exposure profile based on water exchange rate scaled to the average maximum exposure for high (unbroken line) and medium (broken line) concentrations compared with the measured values (squares and circles). Oil addition was terminated after 96 h. (B) Oil droplet size distribution (volumetric) at the inlet of the exposure tank (broken line) and corresponding measurement in the exposure tank (unbroken line graph) and algae mixture used for food (grey, lower left); *x* axis shows particle volume per milliliter of seawater.

The experimental period lasted a total of 26 d, starting with the transfer of 750 young and presumably fertilized female *C. finmarchicus* from the production culture to the 4 exposure tanks (control, low, medium, and high exposure concentrations). Addition of the oil dispersion started the same day that the copepods were transferred to the exposure tanks and continued for 4 d. Oil concentrations in the tanks increased to a maximum value approximately 24 h after oil addition started and approached 0 approximately 24 h after termination of the oil addition (day 5) due to the dilution dynamics of the system (Figure 1A). The exposure duration was expected to be sufficient to approach the incipient median lethal concentration, where the body residue of dissolved oil components is in equilibrium with the water concentration [25; B.H. Hansen, unpublished data]. After termination of exposure the copepods remained in the exposure tanks, now supplied with clean seawater and algae, for another 72 h to allow an initial recovery before being counted. The recorded number of dead animals at this point (day 8) is referred to as acute lethality, and the remaining time period is referred to as the recovery period. The surviving copepods from each exposure concentration were then (on day 8) evenly distributed into 3 recovery tanks for reproduction studies. Nauplii population development in the recovery tanks was monitored daily from day 11 of the experiment. The egg-laying capacity and hatching success of a subset of the test animals was monitored daily for a period of 4 d starting on day 19 (13 d after termination of exposure). Information on the sampling schedule during the experiment is given in Table1.

**Table:** Overview of the timeline of the experiment^a^

	Day																										
	1	2	3	4	5	6	7	8	9	10	11	12	13	14	15	16	17	18	19	20	21	22	23	24	25	26
Copepods kept in exposure tanks	x	x	x	x	x	x	x	x																		
Exposure period	(x)^a^	x	x	x	(x)^a^																					
Transfer of copepods and maintenance in recovery tanks								x	x	x	x	x	x	x	x	x	x	x	x	x	x	x	x	x	x	x
Counting of copepods	x							x																		x
Sampling of offspring in recover tanks											x	x	x	x	x	x	x	x	x	x	x	x	x	x	x	
Single female reproduction tests																			x	x	x	x	x			
Feeding	x	x	x	x	x	x	x	x	x	x	x	x	x	x	x	x	x	x	x	x	x	x	x	x	x	x
Water samples for GC/MS		x		x																						
Particle counting in exposure/recover tanks		x	x	x	x	x						x	x	x	x	x	x		x		x	x				

a Days with increasing or respectively decreasing exposure concentrations.

GC/MS = gas chromatography–mass spectrometry.

#### Exposure tanks

White polyethylene flow-through tanks holding 50 L of water were applied as exposure units. The water level in the tanks was kept stable, and the copepods retained in the system by an outflow device with a large-area 300 µm nylon mesh. Water exchange was adjusted to 70 mL min^−1^ corresponding to 2 exchange volumes per day and a mean water residence time of 12 h by passive regulation of the flow driven by gravity.

Some added turbulence in the tanks was necessary to prevent settling of algae and surfacing of oil droplets and to avoid local oxygen depletion. Because conventional methods (air bubbling, magnetic rod spinning, and recirculation) were excluded due to oil droplet coalescence (or, in the case of magnetic rod spinning, damage to the organisms), a novel stirring method was invented. The method involved an acrylic bowler-shaped dish (150 mm diameter) slowly sliding up and down in the water column along a vertically mounted glass rod inserted through a central bushing in the disc. The upward movement was accomplished by increasing the buoyancy of the dish by filling a built-in chamber with air from a pressurized air source through a polytetrafluoroethylene capillary tube, and regulated by a computerized three-way electric valve (Christian Bürkert & Co. KG). To make the dish sink again, the air was allowed to leak back through the capillary tube and out through the third port of the valve. Computer routines were programmed in LabVIEW (National Instruments Corporation), controlling both the frequency and rate of air filling. One cycle per 10 min proved sufficient for our purposes.

#### Recovery tanks

For recovery, 50 L white polyethylene tanks similar to the exposure tanks were used. Flow regulation, algae addition and feeding rate, as well as water exchange rate were the same as used for the exposure tanks. However, stirring could now be done by gentle air bubbling.

#### Generating exposure solutions and exposure

Crude oil from the Troll B production platform was artificially weathered to 200 °C (200+ residue) through a 1-step distillation procedure 26. In the present experiment, we aimed to keep the concentration of dispersion constant during the experiment by applying the oil droplet generator system 23, which provides a constant supply of dispersions with controlled droplet size distributions and a constant exposure concentration throughout the exposure period. The concentrations of dispersed oil used in the present experiment are comparable to what can be expected in an oil spill situation. The highest concentration (16.5 mg oil L^−1^) is expected to be reached only in the very close vicinity of a surface oil slick or following a deep-sea blowout. The average medium concentration (1.8 mg L^−1^) corresponds to what could be expected in the upper water layer below an oil slick, whereas the highest concentration is above this level 27.

The dispersions were added to the water inlet of the tanks originally filled with clean seawater. The oil concentrations in the tanks thus increased asymptotic toward the maximum value and, according to the exchange rate, should reach approximately 87% of maximum values after 24 h. The addition of oil dispersion continued for a total of 96 h and was then terminated. Due to the water exchange dynamics in the tanks, however, exposure continued for several hours until the oil concentration approached 0 (Figure 1A). The total dose, however, corresponded to 96 h of constant exposure at the maximum level.

#### Single female reproduction test

Ten individuals from each recovery tank were subjected to testing, giving a total of 30 individuals per concentration category. The egg-laying chambers (n =120) for the single female reproduction test (SFR test) were designed for static/semistatic operation. Each chamber was machined from a 75-mm length of a 110-mm black polyethylene pipe, fitted with a 300 µm nylon square mesh stretched over the lower opening. The chamber was placed in a polypropylene food grade container (a 1-L Superfos Unipac), which served as water reservoir and egg collector. A distance of 30 mm between the mesh and container floor was allowed to minimize reflux of eggs to the chamber. To ease handling the units, they were organized in labeled polyethylene boxes, 5 in each, giving a total of 24 boxes.

Approximately 700 mL of filtered seawater with the desired algae concentration (∼2 mg wet wt L^−1^) was added to each unit, leaving an inner water volume of 0.475 L (95 mm × 50 mm) available for the copepod above the mesh. This was assumed to give sufficient space for the comfort of 1 female *C. finmarchicus*. The eggs were expected to fall through the mesh and come to rest on the bottom of the outer container. The shallow water column would restrict the time the eggs would be in the water and hence reduce maternal predation.

Prior to egg collection, the female was transferred to a small glass bowl with seawater using a ladle. The chamber was then lifted out of the outer container, leaving the eggs in the water in the container. The eggs were then concentrated into a glass scintillation vial (8 mL) using a simple custom-made sieving and concentration apparatus. A certain amount of carry-over (1–2 eggs) between subsequent samples was occasionally observed with the use of the apparatus, justifying samples with fewer than 3 eggs being considered naught. After cleaning the container, new seawater with feed algae was added, and the female was returned for the next period of 24 h.

The collected eggs were incubated in the vials at 10 °C to allow hatching. After 48 h, the process was stopped and the material fixed by adding Phytofix (Lugol's solution; ∼20 µL per vial). Unhatched eggs and nauplii could now be identified and counted, still in the vial, using an inverted microscope (Eclipse TE2000S; Nikon).

All individuals involved in the egg-laying test were photographed posttest to verify their gender, developmental status, and their general morphological integrity.

#### Population development in the recovery tanks

A custom-made water sampler (total volume, 165 mL) was used to take daily nauplii samples for estimation of *C. finmarchicus* population development in the recovery tanks during the recovery period. Each day, 5 random subsamples were collected from each tank and pooled, and the copepod material was then collected on a nylon mesh sieve (60 µm square). Before sampling, the water was thoroughly stirred by 2 strokes with a manual version of the stirring device described for the exposure tanks. The collected material was washed into a Petri dish, inspected using a dissecting microscope (MZ125; Leica Microsystems), and pipetted to an 8-mL scintillation glass vial. The material was then fixed as described for egg samples. Later, the samples were stage-determined and counted using the inverted microscope (Eclipse TE2000-S; Nikon).

### Specific analyses

#### Oil in water

Exposure media samples (approximately 900 mL each) for chemical verification of exposure concentrations were collected in baked borosilicate glass bottles and acidified with diluted hydrochloric acid to avoid bacterial degradation. The samples were extracted with dichloromethane, dried over Na_2_SO_4_, and concentrated to 1 mL. Analysis for semivolatile organic compounds—including phenols, naphthalenes, and 3- to 5-ring PAHs—were performed by gas chromatography–mass spectrometry (GC/MS) operated in selected ion monitoring mode. The system was comprised of an Agilent 6890N GC with an Agilent 5975B quadrupole mass-selective detector (MSD). The column was an Agilent J&W HP-5MS fused silica capillary column (60 m 0.25 mm inner diameter × 0.25 µm film thickness). The carrier gas was helium at a constant flow of 1.2 mL min^−1^. A 1-µl sample was injected into a 310 °C split/splitless injector. The oven temperature was programmed from 40 °C for 1 min, then to 315 °C at 6 °C/min and held for 15 min. Data and chromatograms were monitored and calculated using the MSD ChemStation (Ver D.03.00.611) software. The MSD ion source temperature was 230 °C.

#### Particle counting

Particle counting with Coulter Counter (Multisizer 3; Beckman Coulter) was used to prepare the algal mixture used for feeding and to monitor oil droplets and algae in the exposure and recovery tanks. The Coulter Counter was equipped with a cuvette with an aperture of 100 µm. All samples for the measurements were collected in 25 mL polystyrene vials (Kartell), and the oil dispersion samples were analyzed immediately after sampling to avoid loss of droplets due to surfacing. All samples were analyzed with 3 consecutive runs on the Coulter Counter. The results were processed and plotted with the Beckman Coulter particle characterization software (Beckman Coulter, Ver 3.51, 2002 and Ver 4.01, 2008). Calculations of oil droplet concentrations and algae concentrations in the individual tanks were made according to Hansen et al. 1.

#### Fluorescence microscopy

To visualize the presence of oil droplets on and in the organisms, copepods were sampled randomly from the exposure tanks after approximately 72 h of exposure and irreversibly sedated with tricaine methane sulfonate (FinQuel, 1.5 g L^−1^ in seawater; Argent Laboratories). After swimming activity ceased, the animals were transferred to a microscope slide and examined on an inverted microscope (Eclipse TE2000-S; Nikon) equipped with a metal-halide light source (X-cite 120; Lumen Dynamics) and a B2-A filter cube (Nikon). Fluorescent images were captured with a Peltier cooled CCD camera (DS-5Mc with DSU1 Controller; Nikon) operated from a computer running NiS Elements F Ver 2.20 (Nikon).

### Calculations of reproductive endpoints

From the single female reproduction tests, the numbers of offspring (sampled daily for 4 d) were summarized and fecundity calculated (eggs female^−1^ d^−1^). Females that produced 3 eggs or fewer during the 4-d period were classified as “not reproducing” (see *Single female reproduction test*), and percentages of reproducing females were calculated for all treatments. Numbers of eggs and nauplii in the fixated samples from the SFR tests were subsequently counted, and hatching success was determined as the numerical ratio between nauplii and sum of un-hatched eggs and nauplii.

Fecundity of fertile females (offspring female^−1^ day^−1^) in the tanks during recovery was calculated from daily representative samples of NI nauplii as described previously, under the following 3 assumptions: Adult mortality rate (day^−1^) in each tank was constant during the entire recovery period, and applicable to the entire tank population. Durations of the developmental stages were as given by Campbell et al. 22; for example, at 10 °C, the mean duration of nauplii stage 1 (NI) was considered approximately 0.9 d. The parameters generated by the SFR-test (percentage of reproducing females and hatching success) were valid for the entire population in the tank during the whole sampling period.

No relevant data for stage-specific nauplii mortality (including cannibalistic predation) were available and could consequently not be included in the calculations. However, predation on nauplii may be extensive in the applied system; therefore, it could seriously bias the calculation outcome (see *Postexposure reproduction recovery dynamics*).

The time delay from egg-laying to mean stage nauplii NI duration is approximately 1.85 d at 10 °C according to Campbell et al. 22, and fecundity for a specific day was calculated from nauplii collected 2 d thereafter. Hence


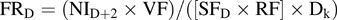


where FR_D_ is the fecundity rate a given day D after transference to the recovery tank; NI_D_ is the number of NI nauplii in the pooled sample from the tank 2 d thereafter; VF is the volume factor: volume_tank_/volume_sample_; SF_D_ is the surviving females in the tank on day D; RF is the fraction of females reproducing, based on the single female reproduction tests and given as the average for females from the 3 replicates; and D_k_ is the average NI stage length (days). Computation of SF_D_ was based on the numbers of females in the tank at the start and end of the recovery period, taking into consideration individuals that were removed (for analyses and single female reproduction test) and assuming a stable exponential population decrease throughout the period.

Both the tank-specific adult mortality rate (percentage per day) and day-by-day population sizes could then be found by iteration of the following expression over the entire recovery period


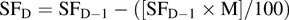


where SF_D_ is the surviving females in the tank at day D, SF_D–1_ is the number of females present the day before day D, and M is the mortality rate.

### Statistical analyses

Statistical analyses and calculation of mean values and standard errors were conducted using GraphPad Prism statistic software, V4.00 (GraphPad Software). Comparison between control groups and exposed groups was performed using analysis of variance (ANOVA) and the Dunnett's post hoc test. For all tests, the level of significance was set at *p* < 0.05 unless otherwise stated.

## RESULTS

### Exposure verification

Oil droplet exposure was verified using the Coulter Counter, and average oil droplet concentrations in individual chambers were recorded daily (Table2). Oil dispersions with nominal concentrations of 25 mg L^−1^, 2.5 mg L^−1^, and 0.25 mg L^−1^ were used for high, medium, and low exposed groups, respectively. As expected, some of the oil was lost, probably due to attachment to surfaces and dissolution and surfacing in the exposure containers. The average volume concentration of oil measured at the inlet of the highest exposure was 21.9 µl L^−1^, whereas the average concentration in the exposure tank with the algae subtracted was 17.9 µl L^−1^ (corresponding to 16.5 mg L^−1^). Figure 1A shows the recorded concentrations and theoretical exposure profile for the high and medium concentrations. The droplet size distribution remained approximately the same in the exposure tanks as in the original dispersion (Figure 1B), with a mean size of 12.0 ± 1.1 µm (mean ± standard deviation [SD]) during the 3 d of maximum concentration (measurements at 4 times). The recorded oil droplet concentrations in the exposure chambers are shown in Table2. The lowest concentration could not be verified directly by particle counting due to low particle density. However, the total oil concentration for this group was estimated based on the relative total concentration of semi-volatile components between the low-exposure group and the medium-exposure group. Concentrations of selected groups of semi-volatile organic compounds are given in Table3. Total concentration of the semi-volatiles in the highest concentration was approximately 0.407 mg L^−1^, of which almost 50% was C0–C4 naphthalenes (0.194 mg L^−1^).

**Table 2:** Oil droplet exposure in mg L^−1^ (assuming density of oil = 0.92 kg L^−1^), measured at daily intervals after start-up of oil addition

Days after start of oil addition					
Group	1	2	3	4	5^a^
High	15.88	15.45	14.86	19.8	2.98
Medium	1.60	1.68	1.82	2.10	0.51
Low^b^	0.019	0.020	0.022	0.025	0.006

a Values in this column represent residual concentrations one day after the oil addition was shut down.

b Calculated from chemical data (total semivolatile organic compounds)

**Table 3:** Summary of chemical analyses of dispersions determined by gas chromatography–mass spectrometry in selected ion monitoring mode (individual component groups)^a^

	Control	Low	Medium	High
Sum all compounds	0.160	0.434	36.2	407
Sum decalins (C0–C4)	0.027	0.013	8.23	102
Sum naphthalenes (C0–C4)	0.089	0.260	18.1	194
Sum phenanthrenes/anthracenes (C0–C4)	0.017	0.067	4.23	46.7
Sum dibenzothiophenes (C0–C4)	<0.001	0.024	1.11	12.0
Sum 2–3 ring PAHs^b^	0.044	0.149	8.41	92.2
Sum 4–6 ring PAHs^c^	<0.001	0.012	1.42	17.7

a Concentrations are given as average (in µg L^−1^) from measurements from days 2 and 4 (n = 2) sampled from individual exposure tanks.

b Includes benzothiophenes, acenaphthylene, acenaphthene, dibenzofurans, fluorenes (C0–C3), phenanthrenes (C0–C4), anthracenes (C0–C4), and dibenzothiophenes (C0–C4).

c Includes fluoranthenes (C0–C3), pyrenes (C0–C3), benz(a)anthracene, chrysenes (C0–C4), benzo(b)fluoranthene, benzo(k)fluoranthene, benzo(e)pyrene, benzo(a)pyrene, perylene, indeno(1,2,3-c,d)pyrene, dibenz(a,h)anthracene, and benzo(g,h,i)perylene.

PAHs = polycyclic aromatic hydrocarbons.

### Feed during recovery

The nominal feed alga concentration in the tanks during the recovery phase with reproduction was 2 mg L^−1^ (wet wt), which corresponded well with the recorded values from the recovery tanks (Supplementary Data S1). The average algae concentration in the 12 tanks ranged from 1.82 mg L^−1^ to 2.23 mg L^−1^, with an overall average of 1.96 ± 0.060 (mean ± SD).

### Fluorescence microscopy

**Figure 2:**
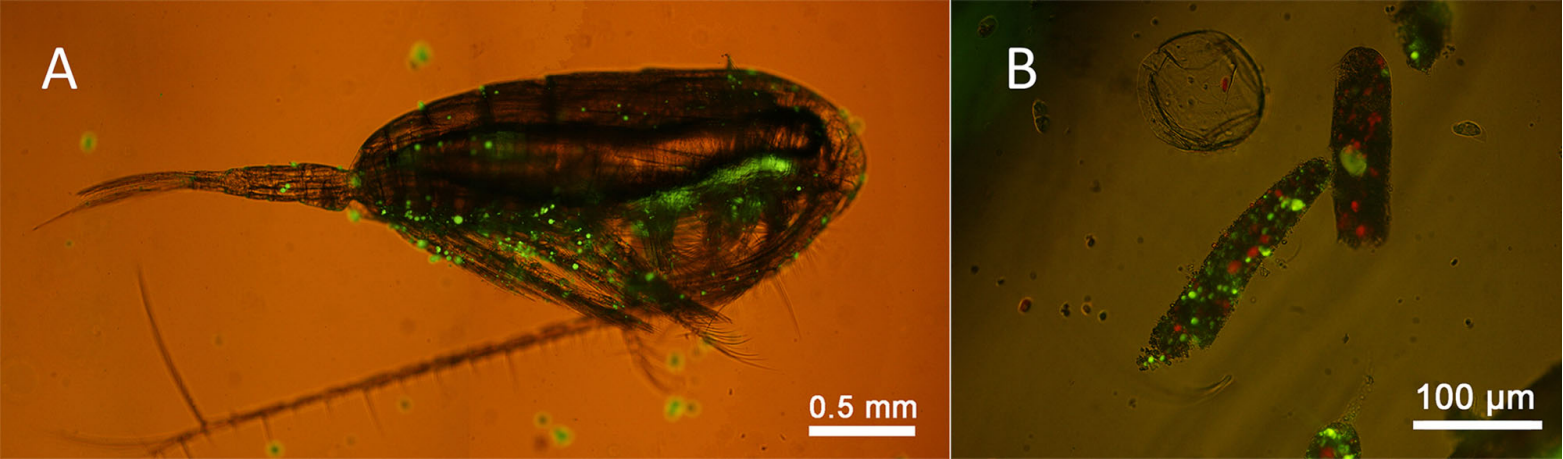
Fluorescence microscopy from medium dispersion concentration. (A) Copepod with oil droplets inside the digestive tract. (B) Fecal pellets containing algae (red) and oil droplets (yellow). Photo Dag Altin.

Fluorescence microscopy revealed oil droplets in the exposure water, on the copepod body surface and in the gut, as well as in fecal pellets. Figure 2A shows a female *C. finmarchicus* from the medium concentration that has filtered and ingested oil droplets. The droplets are visible as yellow spots within the copepod body and on the body surface, while the yellow stains on the lower front of the prosome (main body) are oil droplets that have been filtered from the exposure media and ingested. In Figure 2B, fecal pellets photographed with the same method show both algae (red) and oil droplets (yellow) as clearly visible. Although these observations illustrate the exposure, they are not readily quantifiable.

### Survival in tanks during the experiment

A concentration-dependent decrease in survival was observed after the exposure period. For control, low, medium, and high exposure, 94%, 97%, 85%, and 58% of the copepods survived, respectively. When related to the number of individuals at the start of the recovery period, mortality during the recovery period was also concentration-dependent. The survival during the 16 d in the recovery tanks in control, low, medium, and high exposure was 78.5 ± 9.8%, 79.2 ± 2.9%, 70.9 ± 1.9% and 59.9 ± 8.3% (mean ± SD; n = 3), respectively. The slope of the linear regression between percentage of dead individuals and logarithmic concentration was significantly different from zero (*p* < 0.01).

### Reproduction in tanks during the 21-d recovery period

**Figure 3:**
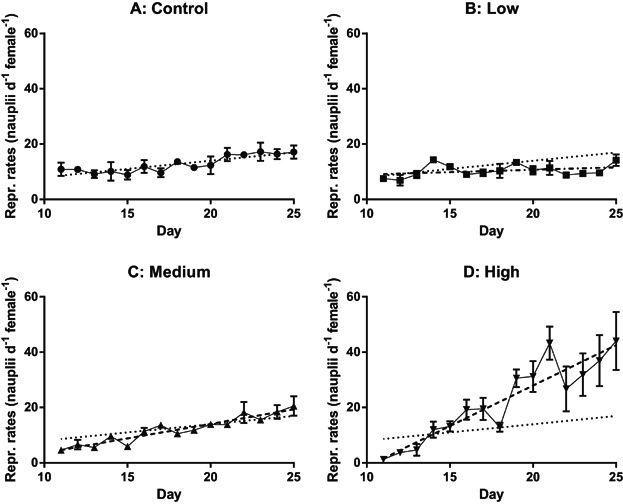
Reproduction rates for all treatments during the whole recovery period (day 11–25) with average ± standard error of the mean from 3 parallel groups from each treatment. Broken and dotted lines indicate the linear regression lines of the data. Dotted line is linear regression line for controls and is repeated for comparison in all plots.

**Figure 4:**
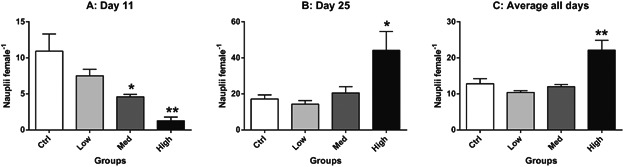
Calculated reproduction rates (offspring female^−1^ day^−1^) in the reproducing fraction of the population (A) at day 11 (5 d after end of exposure; n = 3); (B) at day 25 (19 d after end of exposure; n = 3); and (C) average reproduction rates for the whole period (n = 15, average ± standard error of the mean). Asterisks denote significant differences from controls (*p < 0.05; **p < 0.01). Note the different scales on the *y* axes.

The calculated egg-laying rates based on daily counts of subsamples from all recovery tanks are shown Figure 3. During the first few days, significantly lower rates were calculated for the copepods exposed to medium and high concentrations of dispersed oil, compared with controls (day 11, Figure 4A). During the recovery period, however, this situation was reversed; Egg-laying rates in copepods exposed to the highest dispersion concentration apparently increased beyond any of the other groups from around day 15 (Figure 4C). At day 25 (19 d after the end of exposure), the calculated average number of offspring produced by each female exposed to the highest concentration was more than twice as high as in the other groups.

**Figure 5:**
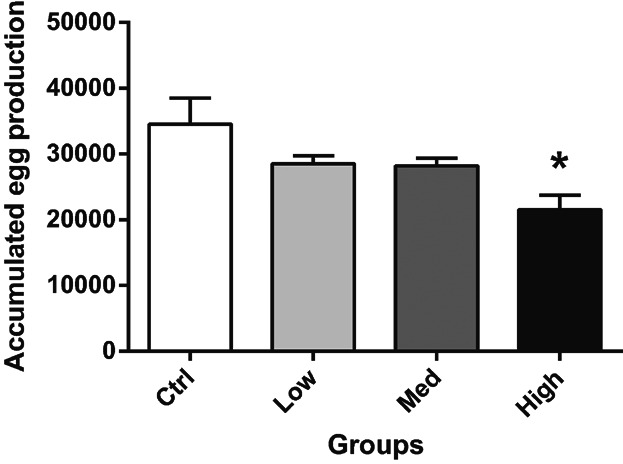
Accumulated number of eggs produced during the whole recovery period (day 11–25) for all treatments. For each group n = 3, average ± standard error of the mean. Initial number of individuals in all groups were 750. Asterisk (*) denotes significant difference from control (*p *< 0.05).

The cumulative numbers of offspring calculated during the whole recovery period (Figure 5) display that, although there was a significant reproductive increase in the group exposed to the highest dispersion concentration, an overall reduction in the number of offspring produced in this group was still observed, compared with the other groups. This relates mainly to lower numbers of females surviving and/or contributing to reproduction.

### Single female reproduction tests

**Figure 6:**
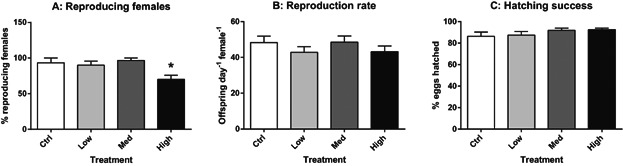
Single female reproduction test: Reproductive parameters for single female copepods exposed to control and 0.22 mg L^−1^ (low), 1.8 mg L^−1^ (medium), and 16.5 mg L^−1^ (high) dispersed oil. (A) Percentage females reproducing during the 4-d reproduction test (n = 3 for all treatments; average ± standard error of the mean); (B) reproduction rate of reproducing females (eggs female^−1^ day^−1^, 4-d test; n = 21–28; average ± standard error of the mean); (C) egg hatching success (percentage of total egg numbers; n = 21–28; average ± standard error of the mean). Asterisk (*) denotes significant difference from controls (*p* < 0.05).

The results from the single female reproduction test show that the number of females contributing to reproduction was significantly lower than control (*p* < 0.05) in the group exposed to the highest dispersion concentration (Figure 6A). Among the females exposed to the highest concentration, 30% did not reproduce, compared with only 3% to 10% in the other groups. After removing nonreproducing females from the calculations, however, no significant differences were found between the exposed copepods and control copepods in terms of egg-laying rate (Figure 6B) and hatching success (Figure 6C).

### Overall loss of reproduction potential

**Figure 7:**
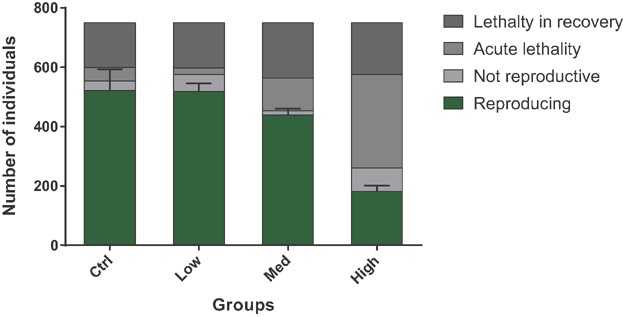
The fate of the initial populations of 750 female *Calanus finmarchicus* after 96-h exposure to different concentrations of dispersed oil. Standard error indicated for reproducing individuals (n = 3). Exposure concentrations were 0 mg oil L^−1^, 0.22 mg oil L^−1^, 1.8 mg oil L^−1^, and 16.5 mg oil L^−1^ for control, low, medium, and high, respectively.

The various impacts on the production of the second generation can be assessed from the previous results. Figure 7 shows the fate of the initial 750 individuals at each level of exposure. The number of individuals lost due to mortality during the recovery period is not different between groups, and the number of individuals not producing eggs is only slightly increased in the high-exposure group. Thus, the dominating factor causing the observed reduction of the number of reproductive individuals in the 2 highest exposures is the acute lethality.

## DISCUSSION

In the present study, a 120-h exposure to dispersed oil resulted in concentration-dependent mortality in *C. finmarchicus* during exposure and recovery. Furthermore, surviving females were monitored for reproduction ability during a 3-wk recovery period. The copepod groups exposed to the medium and high concentration of dispersed oil (1.8 mg L^−1^ oil and 16.5 mg L^−1^ oil, respectively) apparently displayed a short-term decrease in egg-laying rate (female^−1^ day^−1^) relative to the control and low-concentration groups, thus suggesting impaired or delayed reproduction ability. Following a period of recovery, however, the egg production of these groups resumed; and from around day 15 of the recovery period and onward, the calculated egg production rates in the high-exposure group significantly exceeded those of the controls. For the 2 lowest exposures, no significant differences in reproduction rates from controls were found from day 16 onward.

### Single female reproduction test

To study reproduction in recovering females in more detail, the SFR test was run from 11 d into the recovery period on subsets of the copepod groups. As seen in Figure 6, the main exposure effect observed was a significantly lower fraction of females in the highest concentration group participating in egg production. Compared with controls, where 93.3 ± 6.7% (mean ± standard error [SE]) of the females produced eggs, only 70.0 ± 5.8% (mean ± SE) of the females in this group produced eggs during the 4-d period. On the other hand, there were no observed differences in this parameter between controls and the groups exposed to low and medium concentrations. Interestingly, the egg-producing females from the highest exposure concentration group displayed reproduction rates (43.2 ± 3.2 eggs female^−1^ d^−1^ [mean ± SE]) and hatching success (86.3 ± 4.1% eggs hatched of total [mean ± SE]) comparable with those of the controls (48.2 ± 3.5% and 92.6 ± 1.5%, respectively [mean ± SE]). Both egg-laying rates and hatching success are comparable with and generally within the upper range of published numbers for *C. finmarchicus* and other *Calanus* species 28–34. Furthermore, standardized images captured of the individual egg-producing females showed no obvious anomalies in fertile females, again pointing to almost complete recovery of the copepods.

### Postexposure reproduction recovery dynamics

A clear-cut, concentration-dependent depression on reproductive capacity of the exposed groups was demonstrated after terminating the oil exposure. The monitoring of nauplii in the recovery tanks started 5 d after the exposure ended, and the number of nauplii (female^−1^) in the high-exposure groups was still approximately one-tenth of that in the control. The calculated egg production (eggs female^−1^ day^−1^) compared with control was even lower in both the high- (*p* < 0.01) and medium-exposure (*p* < 0.05) groups (Figure 4A), and the swimming behavior of the females seemed affected (more sluggish). The data and observations are comparable with results from a study reported by Jensen et al. 13, in which decreased egg production and hatching success were observed in *C. finmarchicus* exposed to 10 nM and 100 nM of the PAH pyrene, respectively. Jensen and Carroll 16 also found concentration-dependent reduced hatching rates in *Calanus glacialis* Jaschnov exposed to North Sea crude oil water soluble fraction (consisting of 16 PAHs identified by the US Environmental Protection Agency, concentrations ranging 3.6–10.4 µg L^−1^). In contrast, in a study in which the calanoid copepod *Centropages hamatus* (Liljeborg) was exposed to 20 µg L^−1^ to 80 µg L^−1^ dispersed oil for 48 h to 64 h, Cowles and Remillard found no effect on egg production rates but did observe a clear concentration-dependent decrease in egg viability (hatching success) 19. No data for hatching success from the early recovery period in the present study are available, but the difference in egg production response between *C. finmarchicus* in the present study and *C. hamatus* in the referred study 19 may be related to different exposure conditions, oil composition, or species-specific tolerances.

As opposed to most previous studies, in the present study we were able to follow the recovery process over an extended period of time. As noted briefly above, clearly impaired *C. finmarchicus* females in the high- and medium-concentration groups gradually recovered; and after day 17 (11 d postexposure), the females exposed to the highest dispersion concentration had a higher calculated egg production than any of the other groups. This persisted throughout the remainder of the period (up to day 25; Figure 3). The calculated average egg numbers produced per female per day over the entire period was actually significantly higher in the highest exposed group than all the other groups (Figure 4C). This may be explained by a significant compensatory reproductive response in this group after the egg production was temporary depressed (see further discussion). Nevertheless, the total calculated offspring production in the tanks in which females recovered from the highest exposure concentration was still significantly lower than in the control recovery tanks (Figure 5). Hence, the increase in reproduction rate was not sufficient to compensate for the loss of females resulting from acute mortality and/or loss of fertility during exposure and recovery.

Although our argumentation thus far may be essentially valid, the discussion needs some refinement when the calculated egg production numbers from the recovery tanks are compared with the egg production data from the SFR test. For the control and low concentration groups, the calculated daily egg production never exceeded 20 and most of the time remained at a level less than one-fourth of the average daily production revealed from the SFR test (40–50 for all groups; Figure 6B). In addition there was no increase—or at most a weak increase—in the calculated egg production over the recovery period. For the medium-exposure groups, in contrast, the egg production was quite low at the start of the period (approximately 5 per female d^−1^) but increased steadily to approximately 20 at the end of the period. This tendency was even more pronounced for the highest exposed groups, where almost no eggs were produced in the start of the period (Figure 3D), but the production in the last part of the period approached the numbers from the SFR test (Figure 4B).

From the outset, we hypothesized that the control and low-exposure groups would demonstrate egg production capacity similar to the results from the SFR test, whereas the more exposed groups would show dose-dependent reduction in the egg production ability, at least initially. The low initial egg production calculated for the medium- and high-exposure groups seems to support this hypothesis, while the low egg production in the control and low-exposure groups apparently contradicts it. It should, however, be emphasized that the above considerations are based on calculated egg production and not direct egg counts, and the calculations may therefore be inaccurate. The calculations were based on the daily nauplii counts, female counts at the start and stop of the recovery period, and input parameters from the single female reproduction test and from reliable literature (see *Materials and Methods*). But nauplii mortality could not be accounted for, and the calculated egg production actually presupposed 0 mortality.

To counteract both food deficiency and possibly cannibalism, feed algae was added in concentrations that, according to Campbell et al. 22, should be enough for proper egg-laying and nauplii development. The concentration was verified through daily analyses by particle counting. Feeding in all recovery tanks was evident from the presence of fecal pellets on the bottom, but feeding activity could not be measured quantitatively in the applied system. Oil components have been shown to depress feeding activity in copepods 9–35, and reduction in feeding into the first part of the recovery period could very well add to the low initial egg production in the highest exposed groups. However, the SFR test demonstrated egg-laying rates comparable with rates reported as normal for the species 36. Furthermore, because *C. finmarchicus* is an income breeder 22, the feeding ability was probably restored at this time of recovery. We are therefore apt to believe that food scarcity or inadequate feeding can hardly explain the peculiarities in the results.

Although the SFR test indicated normal feeding in all groups in the last part of the recovery period, the calculated egg production in the recovery tanks of the control and low- and medium-concentration groups did not parallel the much higher egg production in the SFR test, despite being fed the same diet. Only the highest exposed groups showed egg production rates similar to the SFR test. Although no irrefutable evidence is available, cannibalistic grazing on nauplii may be involved because both *C. finmarchicus* and relatives are well-recognized cannibals 37–38. For *C. finmarchicus*, Basedow and Tande 37 found very high nauplii clearing rates, averaging to 689 mL female^−1^ d^−1^ and in some cases up to 2 L female^−1^ d^−1^, with only a slight reduction throughout the range 0 L^−1^ to 20 nauplii L^−1^. They also found a linear relationship (measured up to 20 nauplii L^−1^) between nauplii abundance and predation rate (slope, *y* = 0.4338x at 6 °C). Correspondingly, Landry 39 reported nauplii predation rates in *Calanus pacificus* Brodsky that did not level off even at nauplii densities close to 200 L^−1^. Basedow and Tande 37 found no feeding saturation within the applied levels of algae feed, and with particular relevance to the present study, cannibalizing did not seem to be reduced, even at high feed algae concentrations.

Applied to the present study, and considering reproduction dynamics, the predation rate reported by Basedow and Tande 37 would almost triple the calculated egg production rate in the control and low-exposure groups. A certain further upward revision of the predation rate would be necessary, however, to increase the egg production rates to levels revealed from the SFR test. Compared to the study of Basedow and Tande 37, a higher temperature was applied for the present study (10 °C vs 6 °C), and the tanks were rather crowded (up to more than 4 females and 120 N1 + N2 nauplii L^−1^). A further increased predation pressure on the nauplii in the present experiment may therefore be expected.

As opposed to the other groups, the high egg-laying rates in the high-exposure groups in the last part of the recovery period were comparable to the SFR test data, pointing to low nauplii mortality. Also, application of the predation rate from Basedow and Tande 37 to the data from these groups returns unrealistically high egg-laying rates (significantly above SFR test data). The data are hence consistent with low or absent predation in these groups. Also, the high egg-laying rates recorded from the last part of the recovery period indicate sufficient feeding ability.

Based on the above reasoning, the results can be evaluated as follows: 1) egg production rate was acutely affected by the exposure in a dose-dependent manner; 2) during the recovery, a large portion of the exposed females recovered and resumed egg production; 3) the calculated egg production rate of females exposed to the highest concentration was close to actual (SFR test) numbers, possibly due to less nauplii grazing (fewer and/or less viable adults); and 4) the unexpectedly low egg-laying rates calculated for the controls and lower exposures may be explained largely by extensive nauplii grazing.

### Oil exposure considerations

In the present study, fluorescence images provided evidence of filtering, ingestion, and excretion of oil droplets by the female *C. finmarchicus* (Figure 3). This suggests that toxic compounds contained in oil droplets may be bioavailable to the copepods. Previous studies have provided evidence for no or limited contribution of oil droplets to the toxicity of oil dispersions on fish larvae, inferring that the water soluble components cause the majority of toxicity observed 40,41. Copepods, in contrast to fish larvae, actively filter particles from the water phase and could therefore be subject to entry of oil components through the filtering apparatus and intestine 3–43. It has also been debated that uptake of oil components through filtration of contaminated food is a more important transport mechanism than passive diffusion in copepods 13.

### Results considering environmental risk and damage assessment

The main focus of the present study has been the fate of surviving *C. finmarchicus* after an acute oil exposure incident. The rationale behind this approach has been to better understand how endpoints reported from laboratory exposure studies relate to effects on population or ecosystem level. Laboratory studies are used extensively to evaluate environmental impact of pollution, and reported results add to the basic scientific data applied for risk and impact analysis as basis for legislation on, for example, offshore oil emissions and contingency planning.

Most laboratory exposure studies on copepods have so far reported on acute test results or, more generally, results obtained during and/or at termination of exposure. Reported endpoints include lethality 11–14, reproductive effects 19, and recently transcriptional or metabolic markers 2–10. However ecologically relevant, none of these endpoints is easily applicable to calculating long-term impacts on the target species or its natural community without information on recovery ability and life strategies of the species. A population of a species with high recovery ability, high reproduction potential, and corresponding high natural mortality may be more apt to recover quickly after an acute exposure incident. On the other hand, this should not be taken as a general rule. Neither should the recovery ability under laboratory conditions negligently be assumed valid for field conditions without further considerations. Animals surviving short-time exposure apparently unharmed may suffer long-range injuries, and food-web interactions may corrupt the validity of the laboratory results. As an example, enhanced predation on exposed individuals due to temporary impaired mobility may add to population effect.

In the present study, the tested cohort of reproductive *C. finmarchicus* demonstrated a surprisingly high ability to recover from transient high, sublethal exposure to dispersed oil, and the effects of the oil exposure on reproductive output were dominated by acute mortality during and shortly after exposure. Furthermore, the majority of the surviving population eventually reproduced at a normal rate, even after exposures close to the acute median lethal concentration. If verified that the results adequately mirror field conditions, the results hence indicate that risk and impact analysis of oil emissions may be fundamentally based on dose–response relationships from acute toxicity tests with copepods, provided additional lethality during the early recovery period is included. Although the reproduction capacity may prove to recover quickly under field conditions, other potential long-range effects on ecological fitness may significantly add to population impact, such as increased predation due to exposure-related reduction in mobility or simply egg-laying delayed beyond the favorable algal bloom period.

## CONCLUSIONS

Short-term exposure of female *C. finmarchicus* to dispersed weathered crude oil from the Troll offshore oilfield (North Sea) caused concentration-dependent mortality (up to approximately 40%) and a temporary significant postexposure decrease in reproduction rate at the highest dispersion concentration (16.5 mg L^−1^). The reduction in cohort fecundity was probably related to a drop in the number of females actually producing offspring rather than a general reduction in fertility. The females from the highest exposure gradually resumed egg-laying capability during recovery, and a single female egg production test performed from 13 d after the end of the exposure revealed that approximately 70% of the survivors from this group now laid eggs, compared with 93% to 97% for the other exposure concentrations. There were no significant differences in fertility between the groups when the different participation numbers were corrected, and no effects on hatching success were found.

The recovery study of the present experiment demonstrated high recovery ability of *C. finmarchicus*, indicating a modest impact on the population level from delayed effects compared with the acute lethality (presumably by narcosis) occurring during the exposure period.

## Supplemental Data

Figure S1 (197 KB PDF).
