# Supplementary material for: Effects of dispersed oil on reproduction in the cold water copepod Calanus finmarchicus (Gunnerus)
Source: Environ Toxicol Chem. 2013 Jul 16;32(9):2045–55. doi: 10.1002/etc.2273 (PMC3883093; doi:10.1002/etc.2273)
Supplement: Figure S1 — (197 KB PDF). [file etc0032-2045-sd1.pdf]

Supplementory information S1

Feeding during recovery

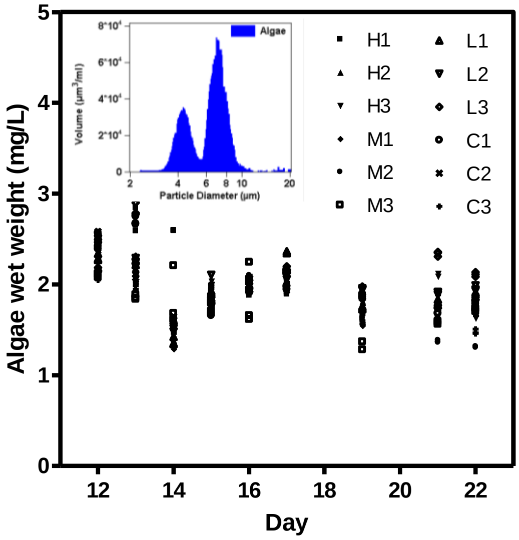

Figure S1. Recorded algal biomass from the recovery containers during the time period where offspring were sampled. H= High exposure groups, M= medium exposure groups, L=low exposure groups and C=control. No measurements were done on 18 and 20. Small figure shows the size distribution of the mixture of algae. X-axis represents days from start of exposure.
